# Judgments at Gaze Value: Gaze Cuing in Banner Advertisements, Its Effect on Attention Allocation and Product Judgments

**DOI:** 10.3389/fpsyg.2017.00881

**Published:** 2017-06-02

**Authors:** Johanna Palcu, Jennifer Sudkamp, Arnd Florack

**Affiliations:** ^1^Department of Psychology, University of ViennaVienna, Austria; ^2^Department of Civil and Transport Engineering, Faculty of Engineering Science and Technology, Norwegian University of Science and TechnologyTrondheim, Norway

**Keywords:** visual attention, online advertising, eye tracking, banner blindness, gaze cuing, animation, advertising effectiveness

## Abstract

Banner advertising is a popular means of promoting products and brands online. Although banner advertisements are often designed to be particularly attention grabbing, they frequently go unnoticed. Applying an eye-tracking procedure, the present research aimed to (a) determine whether presenting human faces (static or animated) in banner advertisements is an adequate tool for capturing consumers’ attention and thus overcoming the frequently observed phenomenon of banner blindness, (b) to examine whether the gaze of a featured face possesses the ability to direct consumers’ attention toward specific elements (i.e., the product) in an advertisement, and (c) to establish whether the gaze direction of an advertised face influences consumers subsequent evaluation of the advertised product. We recorded participants’ eye gaze while they viewed a fictional online shopping page displaying banner advertisements that featured either no human face or a human face that was either static or animated and involved different gaze directions (toward or away from the advertised product). Moreover, we asked participants to subsequently evaluate a set of products, one of which was the product previously featured in the banner advertisement. Results showed that, when advertisements included a human face, participants’ attention was more attracted by and they looked longer at animated compared with static banner advertisements. Moreover, when a face gazed toward the product region, participants’ likelihood of looking at the advertised product increased regardless of whether the face was animated or not. Most important, gaze direction influenced subsequent product evaluations; that is, consumers indicated a higher intention to buy a product when it was previously presented in a banner advertisement that featured a face that gazed toward the product. The results suggest that while animation in banner advertising constitutes a salient feature that captures consumers’ visual attention, gaze cuing can be an effective tool for driving viewers’ attention toward specific elements in the advertisement and even shaping consumers’ intentions to purchase the advertised product.

## Introduction

Online advertisements are often confronted with the problem of being overlooked (Nielsen, 1997). Although such advertisements are usually designed to be particularly attention grabbing, consumers hardly look at banner advertisements on a webpage. This problem is referred to as banner blindness, which was based on the finding that people searching for specific information on a webpage are likely to ignore information that is presented in banners that clearly stand out against other items on the page (Benway, 1998; Drèze and Hussherr, 2003; Burke et al., 2005; Nielsen, 2007a,b).

To address the problem of banner blindness, advertising research is continually exploring new ways to design effective banner ads that can grab consumers’ attention and increase their interest in the advertised products and brands (for a review, see Brajnik and Gabrielli, 2010). One factor that has only recently been suggested to improve banner ad effectiveness is the inclusion of human faces in the advertisement (Sajjacholapunt and Ball, 2014). The rationale behind presenting human faces in banner ads follows three basic assumptions: First, because of their social and emotional significance (Vuilleumier and Schwartz, 2001; Palermo and Rhodes, 2007), faces possess the natural ability to capture an individual’s attention more rapidly and automatically than other nonsocial stimuli (Bindemann et al., 2005; Langton et al., 2006). Consequently, if presented in banner ads, faces are very likely to attract consumers’ attention where other non-social objects or design elements would fail. Second, not only do faces attract attention, but the social signals they convey can direct an observer’s attention toward specific locations in the environment. Specifically, research has demonstrated that a person’s directed gaze can trigger an attentional shift in an observer, “cuing” him or her to look toward the same area or object that the gaze is directed toward (Friesen and Kingstone, 1998; Langton and Bruce, 1999; Frischen et al., 2007). Thus, featuring faces with a certain gaze direction in banner ads might help direct consumers’ attention toward parts of the advertisement they might otherwise ignore (e.g., the advertised product). Third, previous research on the implied social meaning of human gaze suggests that the effect of observed gaze can go beyond merely directing consumers’ attention to a specific object in space but it can also have a sustained impact on the subjective evaluation of the cued object with objects that are looked at usually being evaluated more positively than objects that are not looked at (Bayliss et al., 2006, 2007; Corneille et al., 2009; Bry et al., 2011). Bry et al. (2011) argued that the preference for looked-at objects derives from a mimetic desire in the observer triggered by the belief that individuals typically prefer to look at objects they consider relevant, interesting, or attractive. Hence, displaying models who direct their gaze toward a product in a banner advertisement may be a potentially interesting tool for advertisers to shape consumers’ evaluation of and increase consumers’ interest in the advertised product.

Although the above-mentioned studies illustrate that faces and gaze behavior can be a powerful means for attracting and driving a viewer’s visual attention, surprisingly few studies have investigated these phenomena in an advertising context, and none have examined the evaluative function of observed social gaze. For print advertisements, Hutton and Nolte (2011) demonstrated that participants spent longer looking at advertisements and products when a depicted model’s gaze was directed toward a product compared with when the model’s gaze was directed toward the viewer. A similar study was recently conducted by Droulers and Adil (2015), who found that magazine advertisements featuring a gaze toward a product increased memory performance for the product and the brand. Applying the concept of gaze cuing to online banner ads, Sajjacholapunt and Ball (2014) offered the first support for the usefulness of human faces in overcoming banner blindness. More specifically, the authors demonstrated that banner ads containing human faces were superior in attracting attention compared with banner ads that did not contain human faces. Moreover, visual attention toward the product was higher in advertisements displaying an averted gaze cue toward the product region of the advertisement compared with a direct gaze turned toward the viewer, and memory for information about the brand and the advertising message increased in the averted gaze condition as compared with the direct gaze condition. These results provide the first evidence that human faces in general and human gaze in particular constitute a potentially promising tool for attracting and driving consumers’ visual attention toward banner ads or toward specific content in these ads. However, given that the study by Sajjacholapunt and Ball (2014) is the only currently existing attempt to examine the functionality of social cues in banner ads, further evidence is still needed to validate these findings. Moreover, despite the fact that the effect of social gaze on object evaluations has repeatedly been observed in other research domains applying several object types such as household items or abstract paintings (e.g., Bayliss et al., 2006, 2007; Bry et al., 2011), to the best of our knowledge, no studies have tested the applicability of the gaze cuing effect in an advertising context by examining its impact on critical consumer outcome variables, such as product evaluations or intentions to purchase an advertised product.

Thus, in the present study, we provide an additional test of the applicability of gaze cuing in an online advertisement context. We moreover extend Sajjacholapunt and Ball’s (2014) research and other research on the functionality of social gaze in an advertisement context (Hutton and Nolte, 2011; Droulers and Adil, 2015) and examine whether the gaze direction of a featured model impacts consumers’ evaluations of and intentions to purchase an advertised product. In addition to this, we include an important factor that has not yet been addressed in this context: animation. Animation is a feature that is commonly applied in banner advertisements. Its use, however, is not without controversy. Researchers who tested the hypothesis that animated graphics address humans’ innate predisposition to orientate toward moving objects (Nass and Reeves, 1996) found that animated banner ads enhance visual attention toward the advertised content (Yoo et al., 2004; Hong et al., 2007; Simola et al., 2011; Hamborg et al., 2012) and increase consumers’ clicking behavior (Yoo et al., 2004; Hong et al., 2007). Contrasting theories, however, suggest that, on the basis of past experience, users might have learned to expect that an animated object on a website is a banner advertisement. Consequently, they might employ executive functions that help them avoid orienting toward the source of animation. Following this reasoning and contrary to previous studies that reported positive effects of animation in banner ads, Lee and Ahn (2012) found that animated banners attracted less visual attention than static advertisements, a difference that they assumed was the result of this learned avoidance behavior. Similarly, Pasqualotti and Baccino (2014) supposed that animated advertisements would be more salient and therefore easier to ignore under certain conditions. Sundar and Kim (2005) demonstrated that dynamic advertisements led to improved attitudes toward the advertisement itself but also to simultaneously worse attitudes toward the advertised product compared with static advertisements. The authors argued that dynamic advertisements make an impression on the viewer while the product itself fades into the background. Therefore, the viewer might feel unable to recall enough product information to form an opinion about it. These results imply that consumers may be predisposed to avoid looking at animated banner ads due to their prior experience with these ads, and even if they look at the advertisement, animation may prevent them from attending to the most relevant information in the advertisement (e.g., the product or brand) and may negatively impact their evaluation of the advertised product.

In the present paper, we argue that human gaze is likely to provide a tool that has the potential to help overcome the problems reported for animated banner advertisements: First, given that attention to human faces often operates automatically and unconsciously (Palermo and Rhodes, 2007), it is likely that animated faces can be presented to override consumers’ impulse to ignore animated objects in banner ads and may consequently foster the attention-grabbing function of these objects. Moreover, because faces are rarely perceived to be static elements in real-life environments, moving faces should attract even more attention in banner advertisements than static faces due to their higher ecological validity. Second, instead of distracting consumers from the relevant advertising content, dynamic human gaze may be used to actively guide consumers’ attention toward a desired location in an advertisement. Dynamic gaze movements that first address the observer before turning in a certain direction have been shown to be specifically effective at driving visual attention because they establish a communication link between an observer and the observed person, thus enhancing an observer’s propensity to follow the shifting gaze (Bristow et al., 2007; Senju and Csibra, 2008; Van der Weiden et al., 2010). They are therefore likely to facilitate consumers’ processing of relevant information in an advertisement even more strongly than a statically oriented gaze. Third, in light of the finding that social gaze can impact the evaluation of objects that are looked at (e.g., Bayliss et al., 2006), it is conceivable that faces displaying dynamic gaze movements toward an advertised product counteract the negative consequences of banner animation on product evaluations that have been observed in the past (Sundar and Kim, 2005). In fact, Bry et al. (2011) argue that the effects of gaze direction on object desirability should be highest when individuals are aware of the association between the gaze and the gazed-at object. It is likely that such an association will be highest for dynamic gaze cues that engage the observer before moving toward the object. Consequently, influences of gaze direction on product evaluations may in fact be larger for banner advertisements containing an animated human face than for advertisements featuring static faces.

### The Present Study

To examine whether the concept of gaze cuing constitutes a possible way to increase consumers’ visual attention to banner advertisements in general and targeted elements of the advertisements in particular, we applied an eye-tracking procedure. Specifically, we measured consumers’ eye movements while they viewed a fictitious online shopping page that contained a banner advertisement depicting a product paired with one of the following: a static face gazing toward the product (static-toward), a dynamic face gazing toward the product (dynamic-toward), a static face gazing away from the product (static-away), a dynamic face gazing away from the product (dynamic-away), or no social information at all. All conditions involved the same female face. Moreover, we assessed consumers’ product judgments subsequent to the banner ad exposure in order to examine whether gaze direction and animation impact consumers’ evaluation of (i.e., product attractiveness) and interest in the advertised product (i.e., intention to purchase, willingness to pay for the product).

On the basis of the aforementioned findings, we predicted:

• H1: Banner advertisements that feature a human face attract increased visual attention compared with banner advertisements that do not feature a human face.
• H2: Animated banner advertisements attract increased visual attention compared with static banner advertisements.
• H3: The product area in the banner advertisements attracts increased visual attention when the advertisement features a face looking toward the product compared with when the advertisement features a face looking away from the product (gaze cuing effect).
• H4: The gaze cuing effect is stronger for dynamic gaze cues than static gaze cues.
• H5: Subsequent product judgments (i.e., product attractiveness, intentions to purchase, willingness to pay) are higher when the product advertisement features a face looking toward the product compared with when the advertisement features a face looking away from the product (gaze cuing effect on product judgments).
• H6: The gaze cuing effect on product judgments is stronger for dynamic gaze cues than static gaze cues.

## Materials and Methods

### Participants and Design

One hundred and thirty-seven participants (94 women, *M*_age_ = 23.66 years, *SD*_age_ = 9.65 years) participated in an eye-tracking experiment for either course credit or monetary compensation (approximately $4 US). The total sample had an above-average level of education: 0.7% had primary, 70.6% had secondary, and 27.9% had higher education. The majority of participants (87.5%) indicated that they frequently buy products online (at least more than once a year). The other participants (12.5%) indicated that they buy products online never or less than once a year. Overall, 56.2% of the participants stated that they usually use an advertisement blocker when browsing the Internet.

A 2 × 2 between-subjects design with the factors gaze direction (toward vs. away from product) and animation (static vs. dynamic face) was applied. In addition, we included a control condition depicting a banner ad without a human face in order to examine whether advertisements containing a human face are superior at attracting consumers’ attention over advertisements containing no social information.

### Apparatus and Stimuli

Eye-tracking data were collected with the remote Eye-tracker SMI RED 500 (Sensomotoric Instruments GmbH, Teltow, Germany) at a sampling rate of 120 Hz. This device allows for head movements within an imaginary head box of about 20 inches × 12 inches. Ambient conditions (e.g., noise and lighting) were held constant during the experiment. All instructions and stimuli were presented on a computer screen that had a width of 22 inches and a resolution of 1680 pixels × 1050 pixels. Participants were seated at a viewing distance of approximately 60 cm from the computer screen.

### Website

**FIGURE 1:**
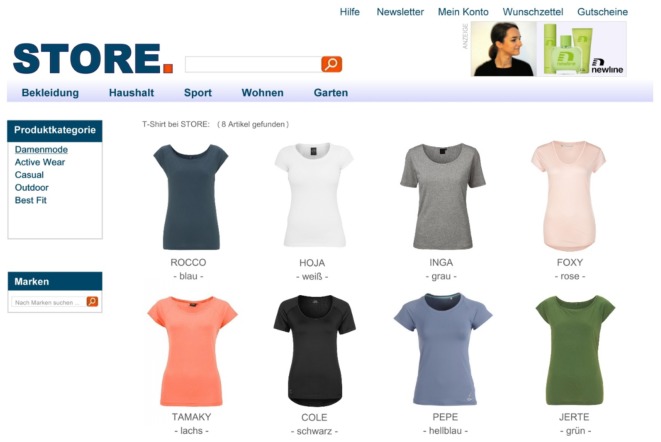
Third webpage used in the experiment with static-toward target advertisement.

**FIGURE 2:**
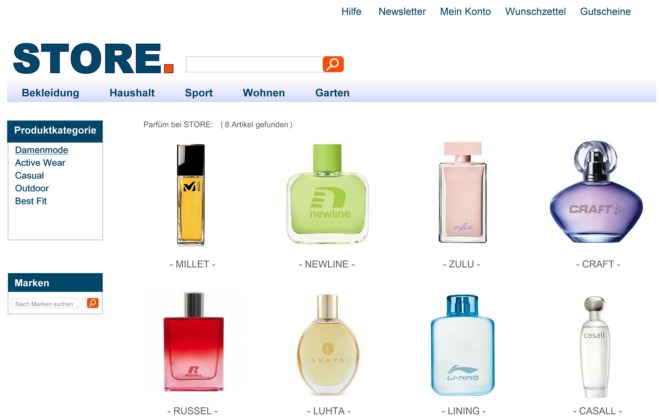
Fourth webpage used in the experiment with the target product in the second place of the first row.

We constructed a fictional online shopping site. The website was designed to resemble an authentic online store. It contained common elements such as the logo of the online shop, navigation bars, search fields, etc. Each participant was presented a total of four different webpages. On each webpage, six to eight different products from a certain product category (i.e., handbags, jackets, shirts, and perfume bottles) were displayed in the lower center of the webpage. All webpages were presented in a fixed order: First, participants were presented two filler pages (i.e., displaying handbags and jackets, respectively) to familiarize them with the design of the website and the experimental procedure. The third webpage (i.e., displaying shirts) contained the target banner advertisement (i.e., perfume) in the upper right corner (**Figure 1**). The fourth and final webpage displayed eight different fictitious perfume brands of which the second product in the first row was the target perfume advertised in the previous trial (**Figure 2**).

A pretest (*N* = 18) revealed that all presented perfumes and brand logos were rated average in terms of attractiveness (*M*_Brands_ = 3.45, *SD*_Brands_ = 1.51; *M*_Products_ = 3.79, *SD*_Products_ = 1.68; all *p*s > 0.05).

### Banner Advertisements

We created vertical banner advertisements (312 pixels × 104 pixels) for a fictitious perfume set. The product along with a brand name was placed on the right half of the banner. On the left half, four of the five experimental groups saw a female face displaying a moderately positive facial expression. In two conditions, the face looked toward the perfume set. In the other two conditions, the model’s gaze was turned away from the advertised product. In the static conditions, the face was presented as a still image looking either toward or away from the advertised product. In the dynamic conditions, the advertisement showed a fluently moving face: The face first looked straight toward the viewer and then slowly turned her head either toward or away from the product, afterward returning to the starting position. Participants in the control group saw only the product without a face.

**FIGURE 3:**
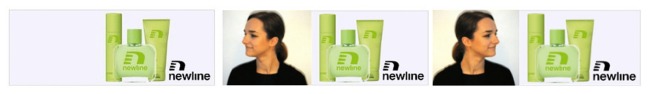
Examples of static banner advertisements as seen by the participants in the no-face, static-toward, and static-away conditions.

All banner advertisements were integrated as GIF files and placed in the upper right corner of the webpage. **Figure 3** provides samples of the different advertisements used in the experiment for the control and static conditions.

### Procedure

Prior to the experiment, participants gave written informed consent and were briefly told about the procedure and the noninvasive nature of the eye-tracking measurement. After the eye-tracking device was calibrated, participants were given the task of purchasing a gift for a female friend from an online shop. They were then presented four different webpages that each displayed a number of products from one product category (i.e., handbags, jackets, shirts, perfume bottles; in order of presentation). Each webpage was presented for 15 s followed by the task of using a 7-point scale to sequentially rate the attractiveness of each of the displayed products (“How attractive do you find this product?”; 1 = *not at all attractive* to 7 = *very attractive*), participants’ likelihood of purchasing the product for a friend (“How likely would you be to purchase this product for your friend?”; 1 = *not at all likely* to 7 = *very likely*), and their willingness to pay for the product (“How much would you be willing to pay for this product compared with the other products in this category?” 1 = *much less* to 7 = *much more*). Verbal reporting was used to avoid disrupting the eye-tracking measurement. Answers were recorded by the experimenter. After participants completed the two filler trials, the webpage with the target banner advertisement was displayed, featuring either a static-toward, static-away, dynamic-toward, dynamic-away, or no-gaze cue. Again, participants rated the products after viewing them (i.e., shirts). The final trial presented the advertised perfume in the array of similar products, again followed by the ratings of all perfumes.

Following the eye-tracking procedure, participants completed a questionnaire on their demographic data on a separate laptop. In addition, a recognition task was conducted in which participants were asked to indicate whether they had seen any product more than once during the webpage presentations. If participants answered “yes,” they were presented six different products, including the target perfume, and were asked to choose the ones they remembered seeing more than once. They were then debriefed and thanked for their participation.

### Ethics Statement

According to the Austrian Universities Act 2002 UG2002 [Universities Act (UG) BGBl. I No. 120/2002], which was in place at the time the study was carried out, only medical universities were required to appoint ethics committees for clinical tests, application of medical methods, and applied medical research. Consequently, no ethical approval for this specific study was required.

The present study was conducted in accordance with the Declaration of Helsinki (revised 1983) and local guidelines of the Faculty of Psychology, University of Vienna. All participants gave written informed consent. Participants could withdraw at any time during the experiment without further consequences. At the end of the experiment, participants received a detailed debriefing.

## Results

Eye movement data and attention maps were computed with the aid of SMI BeGaze Analysis software (version 3.4 SMI, 2013). All analyses were performed in SPSS. We analyzed participants’ gaze data for three predefined areas of interests (AOIs): the banner advertisements as a whole (banner AOI), the region of the advertisement depicting the human face (face AOI), and the region showing the advertised product (product AOI). All AOIs were examined with regard to two key attention measures: the consumers’ likelihood of attending to the AOI (AOI hit likelihood; dummy coded as 0 and 1) and the overall time they spent gazing at the AOI (AOI dwell time; in ms).

### Preliminary Analyses

We tested our dwell time data for skewness and deviations from a normal distribution. Dwell time data for all AOIs were positively skewed (≤4.560) and showed significant deviations from normality [*D*s (137) ≤ 0.346, *p*s < 0.001], which is a very common observation for biometric gaze data (Holmqvist et al., 2011, p. 387). To account for this deviation from normality, we implemented a generalized linear model (GZLM; Nelder and Wedderburn, 1972) for compound poisson-gamma-distributed dependent variables for all analyses that included dwell time as the dependent variable. This model assumes a continuous gamma distribution for the dwell time data while simultaneously accounting for the non-occurrence of events indicated by dwell time values of zero (Jørgensen, 1997).

### Effects of Static and Animated Human Faces on Attention to Banner Advertisements

We hypothesized that the depiction of a human face in a banner advertisement would result in an increase in consumers’ likelihood of attending to the advertisement (H1). To test our prediction, we compared banner AOI hit likelihood (dummy coded as 0 and 1) for the advertisement that contained a static human face with the banner AOI hit likelihood for the baseline condition that had no human face. Contrary to our expectations, results indicated that including a human face did not increase consumers’ likelihood of attending to the banner advertisement, χ^2^(1, *N* = 84) = 0.979, *p* = 0.323.

We moreover expected that a banner advertisement with an animated face would attract more attention than a banner advertisement that displayed a static face or no face at all (H2). In line with our expectations, we found a marginally significant difference when comparing the banner AOI hit likelihoods for static and animated banner advertisements containing a face, χ^2^(1, *N* = 108) = 3.315, *p* = 0.069, showing that animated faces were 2.073 times more likely to direct participants’ attention to the advertisement than static faces. Comparing advertisements depicting an animated human face with the baseline condition that had no human face also yielded a significant difference in the banner AOI hit likelihood, χ^2^(1, *N* = 82) = 6.301, *p* = 0.012. Specifically, the odds ratio for an AOI hit on the banner advertisement was 3.276 times higher when the advertisement featured an animated face than when it contained no face at all. In summary, these results suggest that banner advertisements that involve a human face are only superior to advertisements without a human face when the featured face is animated but not when it is static.

To examine whether the depiction of a static, animated, or no human face had an influence on the time consumers spent looking at the advertisement, we computed a generalized linear model (GENLIN in SPSS 20) with the time spent dwelling on the banner AOI as a poisson-gamma-distributed dependent variable and the banner advertisement type (static, animated, or no face) as the independent variable. Results indicated that the banner advertisement type significantly impacted the time participants spent dwelling on the banner advertisement, Wald χ^2^(2, *N* = 137) = 16.540, *p* < 0.001. *Post hoc* pairwise comparisons at each level of the banner advertisement type revealed that participants looked significantly longer at the banner advertisement when it contained an animated face than when it contained a static face [*M*_animated face_ = 995.94 ms, *SD*_animated face_ = 1496.20 ms, *M*_static face_ = 253.87 ms, *SD*_static face_ = 463.34 ms, *t*(106) = 4.006, *p* < 0.001; Bonferroni corrected]. Moreover, the banner advertisement featuring an animated face had a higher dwell time than the advertisement without a human face [*M*_animated face_ = 995.94 ms, *SD*_animated face_ = 1496.20 ms, *M*_no face_ = 262.37 ms, *SD*_no face_ = 715.86 ms, *t*(80) = 3.759, *p* = 0.001; Bonferroni corrected]. Dwell times for the static face and the no face advertisements were not significantly different, *p* > 0.10. These findings indicate that advertisements presenting moving social stimuli show an advantage in retaining attention over advertisements that present a static face or no social information at all.

### Effects of Gaze Direction and Face Animation on Attention to Advertised Products

On the basis of the attention cuing effect of human gaze that has been described in previous literature (for an overview, see Frischen et al., 2007), we predicted that the product area within a banner advertisement would attract and retain more attention when the advertisement depicted a face gazing at a product than when it contained a face gazing away from the product (H3). Moreover, we expected that this gaze cuing effect would be stronger for animated than static faces due to the higher ecological validity of animated faces (H4). So that we could test the gaze cuing effect, the following analyses included only the participants who initially gazed at the banner advertisement (*N* = 78).

**FIGURE 4:**
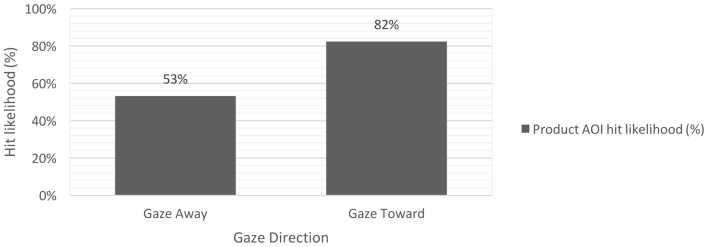
AOI hit likelihood on product AOI in the gaze direction conditions (gaze toward vs. gaze away from product AOI).

To test our hypotheses, we first computed a binary logistic regression with the product AOI hit likelihood (dummy coded as 0 and 1) as the dependent variable and the factors animation (static vs. animated) and gaze direction (gaze toward vs. away from product) as predictors, χ^2^(3, *N* = 78) = 8.478, *p* = 0.037, Nagelkerke’s *R*^2^ = 0.169. Results indicated a significant main effect of gaze direction in predicting the product AOI hit likelihood, Wald *z* = 6.757, *p* = 0.009 (**Figure 4**). The odds ratio indicated that the odds for an AOI hit on the product area were 4.117 times higher when the gaze was directed toward (as compared with away from) the product. The main effect of animation was not significant, Wald *z* < 0.10, *p* > 0.10. Contrary to our expectations, the gaze cuing effect was not stronger for animated than static faces as indicated by the non-significant animation by gaze direction interaction, Wald *z* = 1.724, *p* > 0.10. Overall, these findings partially confirmed our hypotheses, indicating that gaze can direct attention toward specific elements in an advertisement (confirming H3) independent of whether the gaze is animated or not (disconfirming H4).

To test whether gaze direction and animation had an effect on the time participants spent looking at the product AOI, we computed a generalized linear model with the time spent dwelling on the product AOI as a poisson-gamma-distributed dependent variable and the factors gaze direction and animation as independent variables. Neither animation nor gaze direction nor the interaction of animation and gaze direction had a significant effect on the time participants spent dwelling on the product AOI (*p* > 0.10), suggesting that neither the animation nor the gaze direction of the featured face had an impact on the time consumers spent examining the advertised product.

### Additional Analysis: Effects of Gaze Direction and Face Animation on Attention Allocation between Face and Product

It is interesting that our results indicate that, while animated faces (compared with static faces) increase the time consumers spend looking at banner advertisements, animation does not seem to increase the time consumers spend dwelling on the advertised product, not even when the animated advertisement features a face that is looking toward the product region of the advertisement. One explanation for this discrepancy could lie in the inherent tendency of moving elements to capture individuals’ attention and distract them from looking at other simultaneously presented elements (cf. Sundar and Kim, 2005). In other words, it is possible that the animated faces in the banner advertisement engaged participants’ attention to such an extent that they ignored the advertised product regardless of where the face was looking.

To test this idea and examine the differences in dwell time between the face and the product region of the banner advertisement, we computed a generalized estimated equation model (GEE) with the between-subjects factors animation and gaze direction and the within-subject factor AOI (product AOI vs. face AOI). Similar to the GZLM, the GEE adopts a compound poisson-gamma distribution for the dependent variable dwell time but it additionally allows for the specification of a within-subject factor.

**FIGURE 5:**
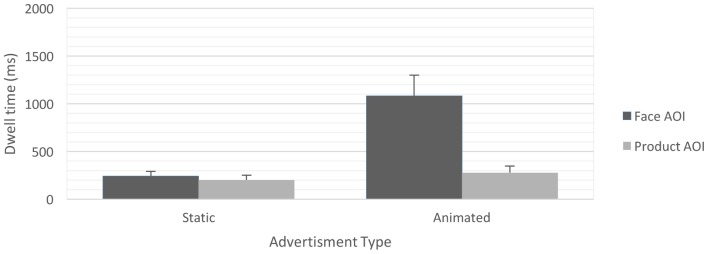
Mean time spent dwelling on face and product AOI for banner advertisements with static as compared with animated faces. Error bars represent the standard errors of the mean.

**FIGURE 6:**
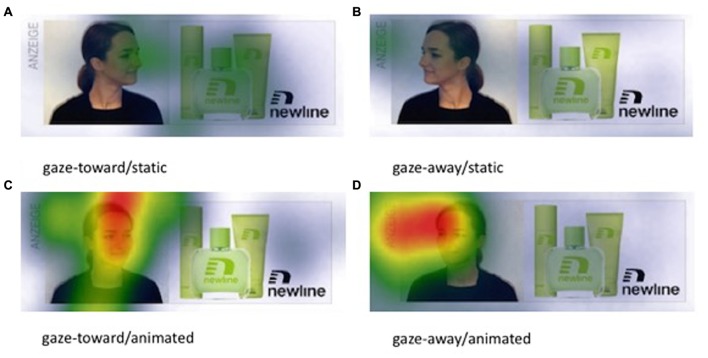
Heat maps showing the accumulated time participants spent looking at different areas of the banner advertisement for the four experimental conditions **(A–D)** summarized across all participants who initially gazed at the banner advertisement. Images are scaled by fixation duration relative to all other elements of the web page and display a 3-color coding ranging from blue (min. fixation duration) to red (max. fixation duration).

There was a significant main effect of the AOI, Wald χ^2^(1, *N* = 66) = 14.491, *p* < 0.001, indicating that, overall, participants paid significantly more attention to the face AOI than to the product AOI [*M*_face_ = 669.70 ms, *SD*_face_ = 1053.33 ms, *M*_product_ = 269.70 ms, *SD*_product_ = 480.77 ms, *t*(65) = 3.807, *p* < 0.001; Bonferroni adjusted]. We additionally found a significant main effect of animation, Wald χ^2^(1, *N* = 66) = 10.859, *p* = 0.001, again indicating that animated banner advertisements received more attention than static advertisements [*M*_animated_ = 681.39 ms, *SD*_animated_ = 773.50 ms, *M*_static_ = 222.41 ms, *SD*_static_ = 226.36 ms, *t*(64) = 3.295, *p* = 0.002; Bonferroni adjusted]. Moreover, we found a significant two-way interaction of the factors AOI and animation (**Figures 5**, **6**), Wald χ^2^(1, *N* = 66) = 10.646, *p* = 0.001. *Post hoc* pairwise mean comparisons revealed that, for advertisements featuring animated faces, the face region received significantly more attention than the product region [*M*_animated face_ = 1083.57 ms, *SD*_animated face_ = 1368.50 ms, *M*_animated product_ = 279.20 ms, *SD*_animated product_ = 446.75 ms, *t*(36) = 3.641, *p* = 0.002; Bonferroni adjusted]. Moreover, animated faces received more attention than static faces [*M*_animated face_ = 1083.57 ms, *SD*_animated face_ = 1368.50 ms, *M*_static face_ = 242.86 ms, *SD*_static face_ = 334.36 ms, *t*(64) = 3.529, *p* = 0.003; Bonferroni adjusted] and the products presented with the static faces [*M*_animated face_ = 1083.57 ms, *SD*_animated face_ = 1368.50 ms, *M*_static_ _product_= 201.97 ms, *SD*_static product_= 288.46 ms, *t*(64) = 3.829, *p* = 0.001; Bonferroni adjusted]. Pairwise comparisons at other levels of the conditions were non-significant (*p*s > 0.10). All other main effects and interactions were non-significant (*p*s > 0.10).

### Effects of Gaze Direction and Face Animation on Product Evaluation

In line with the previously reported positive effects of directed gaze on evaluations of gazed-at objects (e.g., Bayliss et al., 2006), we expected product evaluations to be higher when the banner advertisement featured a gaze that was turned toward (vs. away from) the advertised product (H5). Moreover, we assumed that a dynamic gaze that engaged the consumer before turning toward the advertised product would enhance the evaluation of the looked-at product even more than a static gaze (H6).

To test our hypothesis, we first computed a one-way ANOVA with the banner advertisement type (static-toward, static-away, dynamic-toward, dynamic-away, and no face) as the independent variable and product attractiveness, intention to purchase the product, and willingness to pay for the product as dependent variables. Results revealed that product attractiveness and willingness to pay were not significantly influenced by the experimental conditions (*p*s > 0.10). Banner advertisement type did, however, influence participants’ intentions to buy the advertised product, *F*(4,73) = 3.094, *p* = 0.021, = 0.145. Means and standard deviations for the intentions to purchase the advertised product in the different experimental conditions are depicted in **Table 1**.

**Table 1:** Intentions to purchase the advertised product for experimental conditions.

	Experimental condition									
	Static						Animated			
	No face		Gaze toward		Gaze away		Gaze toward		Gaze away	
	*M*	*SD*	*M*	*SD*	*M*	*SD*	*M*	*SD*	*M*	*SD*
Purchase intention Target product	3.50^a^	1.83	3.18^b^	1.70	2.42	1.24	2.41^a^	1.23	1.90^a,b^	1.53

*^a,b^indicate significant post hoc group mean comparisons at p < 0.05.*

To examine this effect in more detail, we computed separate 2 × 2 ANOVAs with gaze direction, animation, and the interaction of animation and gaze direction as independent variables and product attractiveness, intention to purchase the product, and willingness to pay for the product as dependent variables. Results for the product attractiveness ratings revealed a marginally significant main effect of animation on product attractiveness, *F*(1,62) = 3.764, *p* = 0.057, $\eta_{p}^{2}$ = 0.057. *Post hoc* mean comparisons indicated that products advertised in animated banner advertisements were perceived to be slightly less attractive than products that were displayed in static banner advertisements [*M*_animated_ = 2.62, *SD*_animated_ = 1.34, *M*_static_ = 3.34, *SD*_static_ = 1.63, *t*(64) = 1.930, *p* = 0.059]. Contrary to our expectations, neither gaze direction nor the gaze direction by animation interaction yielded significant results on product attractiveness (*F*s < 1, *p*s > 0.10).

**FIGURE 7:**
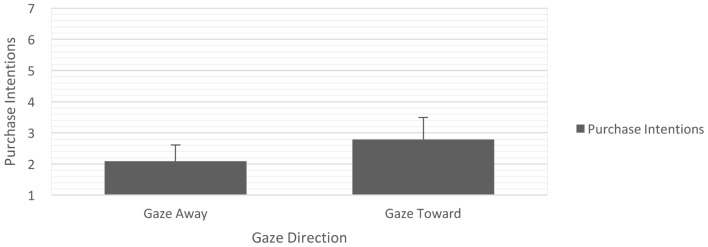
Mean intentions to purchase advertised product for banner advertisements with gaze toward as compared with gaze away from advertised product. Error bars represent the standard errors of the mean.

Results for consumers’ intentions to purchase the target product revealed a significant main effect of gaze direction on purchase intentions, *F*(1,62) = 3.405, *p* = 0.035, $\eta_{p}^{2}$ = 0.026, one-tailed (**Figure 7**). In line with our expectations, we found that purchase intentions were higher for products that were displayed with a face gazing toward (vs. away from) the product [*M*_toward_ = 2.79, *SD*_toward_ = 1.51, *M*_away_ = 2.09, *SD*_away_ = 1.25, *t*(64) = 2.041, *p* = 0.045]. Additionally, we found a marginally significant main effect of animation on consumers’ intentions to purchase the target product, *F*(1,62) = 3.458, *p* = 0.068, $\eta_{p}^{2}$ = 0.053. *Post hoc* mean comparisons indicated that consumers’ intentions to purchase the advertised product were lower for animated than static banner advertisements [*M*_animated_ = 2.14, *SD*_animated_ = 1.25, *M*_static_ = 3.34, *SD*_static_ = 1.63, *t*(64) = 2.108, *p* = 0.039]. The gaze direction by animation interaction was not significant (*F* < 1, *p* > 0.10).

Finally, consumers’ willingness to pay was not influenced by animation, gaze direction, or the animation by gaze direction interaction (*p*s > 0.10).

Overall, our findings imply that while gaze direction influences consumers’ intentions to purchase a product, attractiveness and the willingness to pay for the product remain unaffected by the gazing behavior of the featured face (partially confirming H5). Moreover, contrary to our expectations, animation did not bolster the gaze cuing effect on product evaluations but rather seemed to have an overall negative impact on consumers’ product judgments (disconfirming H6).

## Discussion

Despite the well-established attention-grabbing and attention-directing functions of human gaze, surprisingly few studies have investigated its applicability in an advertising context in general (Hutton and Nolte, 2011; Droulers and Adil, 2015) and in an online advertising context in particular (Sajjacholapunt and Ball, 2014), all demonstrating a positive influence of perceived gaze shifts on consumers’ attention to gazed-at objects in an advertisement. While these findings are very promising, they have failed to address the evaluative importance of gaze information even though the positive impact of gaze on the evaluation of gazed-at objects has repeatedly been observed in previous studies (Bayliss et al., 2006, 2007; Corneille et al., 2009; Van der Weiden et al., 2010; Bry et al., 2011). Evaluative effects of gaze are, however, of major significance in an advertising context where positive evaluations of and interest in the advertised product are typically the primary goal. Applying an eye-tracking procedure, the present research therefore aimed to (a) offer support for the importance of human faces and their gazing behavior for consumers’ visual attention to banner advertisements and their evaluation of the advertised products (b) extend prior findings by including an additional factor that has been the subject of recent debates in the banner advertisement literature: animation.

Contrary to our expectations and the research by Sajjacholapunt and Ball (2014), we found that the mere presence of a (static) face in a banner advertisement did not increase consumers’ probability of looking at the advertisement and the time they spent evaluating that advertisement during their online webpage exploration. There are a few factors that may explain this deviation from Sajjacholapunt and Ball’s (2014) original findings. First, the banner advertisement that we used was smaller in size than the advertisements that were used originally. Thus, participants might have shown an overall higher tendency to overlook our advertisement regardless of whether it included a face or not. Second, despite the important social and emotional functions that faces and the information they convey serve in everyday life (Vuilleumier and Schwartz, 2001; Palermo and Rhodes, 2007), consumers might have learned, through frequent exposure to advertisements featuring social stimuli, to automatically ignore faces in an online context where advertising clutter is expected to be high and advertisements are perceived to be particularly intrusive (Li et al., 2002). Consequently, due to the small size and the peripheral position of the advertisement in our experiment, it might have been easier for consumers to inhibit their initial reflex to look at the advertisement featuring a static human face.

Instead our results show that animated faces are superior to static faces in directing and retaining consumers’ attention to banner advertisements. Animation is frequently applied in online advertisements as a means for grabbing consumers’ attention (Yoo et al., 2004; Hong et al., 2007; Simola et al., 2011; Hamborg et al., 2012). Its effectiveness, however, has been strongly debated in the literature, with recent studies showing that animated banners can in some cases even receive less attention than static banner advertisements (Lee and Ahn, 2012; Pasqualotti and Baccino, 2014). Our results suggest that, at least for animated advertisements containing human faces, banner blindness is less likely to occur. Overall, our results imply that consumers look more frequently at advertisements and engage more with the advertisement when it incorporates an animated face. While these results might seem to suggest that animation can increase ad perception and ad content processing for consumers who look at an advertisement, our further analyses revealed that it is mainly the animated object that profits from the animation (in this case, the face), while other elements of the banner advertisement, such as the product, do not receive increased visual attention. These findings corroborate previous observations that animation may induce less interest in the animated product. For example, Sundar and Kim (2005) demonstrated that animation can improve attitudes toward the advertisement itself but can simultaneously lessen involvement with the advertised product. They argued that animation as a salient feature of an advertisement distracts the viewer from the actual information that is presented. As a consequence, while viewers engage more with the advertisement itself, product involvement as well as product knowledge and intention to purchase decline.

Finally, we found that participants who looked at advertisements displaying a face that was turned toward the advertised product were more likely to look at the product area than participants who saw advertisements displaying a face that was turned away from the product. This is in line with previous research that suggested that the observation of gaze cuing leads to an attentional shift toward the looked-at region (Hutton and Nolte, 2011; Sajjacholapunt and Ball, 2014; Droulers and Adil, 2015). Increasing the likelihood that a product will be noticed already constitutes an important first step for advertisements to arouse a consumer’s interest. However, even though participants in the present study were more likely to look at the product region when the region was gazed-at, they did not look longer toward the respective area compared with participants who observed a face that was turned away from the product. While this might seem to contradict the results from previous studies on gaze cuing in an advertising context, it is important to point out that the methodological procedure of the present study differed from the approach used in previous research. Hutton and Nolte (2011) as well as Sajjacholapunt and Ball (2014) compared the influence of a face looking at a product versus a face looking directly at the viewer. Their procedure was based on the assumption that faces directing their gaze toward an observer are more efficient at capturing attention than faces displaying an averted gaze (Frischen et al., 2007). However, in order to be able to display a similar degree of animation for the different levels of gaze direction, in the present study, we varied whether the model turned toward or away from the advertised product. No condition was applied in which the advertisement featured a model looking straight ahead. Hence, while gaze cues directed at the product seem to be advantageous over direct gazes (i.e., looking at the observer) in influencing consumers’ engagement with a product, the results of the present study do not provide evidence that gaze cues turned toward a product are also advantageous over gaze cues directed away from the product. Furthermore, in contrast to the present study, the above-mentioned studies did not restrict the time for which the advertisements were displayed. In the study by Hutton and Nolte (2011), participants browsed through a screen magazine and were free to turn the pages forward or backward at any time. In the study by Sajjacholapunt and Ball (2014), participants were given the task of searching for specific information on different websites, and they could move on to another webpage independently. In the present study, the display time of each webpage was limited to 15 s. This allowed for a more controlled measurement of consumers’ engagement with online advertisements. Even though we acknowledge that, in an authentic online context, the exposure time varies widely, user behavior studies have suggested that on average users spend between only 10 to 20 s on a webpage (Nielsen, 2011). Although the average time spent on a page differs between different types of websites, these findings clearly suggest that advertisers have only a limited amount of time to grab a viewer’s attention. In terms of practical implications, gaze cuing effects that occur within this limited time span are therefore of particular relevance.

Most interesting, however, although the effects of gaze cuing did not result in an enhanced visual processing of the advertised product, an effect of gaze direction was observed on the subsequent evaluation of the product when the product was presented within an array of similar products subsequent to the ad exposure. More specifically, participants indicated a higher intention to purchase the advertised product when the model’s gaze was turned toward (vs. away from) the product. However, product attractiveness ratings and willingness to pay remained unaffected by the gaze direction of the featured face. The fact that gaze direction did not impact product evaluations (e.g., product attractiveness ratings) seems somewhat surprising, given the previous observation that objects that are being looked at by a neutral or smiling face appear to be more likable than objects that are not being looked at (Bayliss et al., 2006, 2007). However, recent findings suggest that the effect of gaze is considerably weakened for valenced stimuli (e.g., other faces) because these stimuli automatically elicit affective responses that cannot be easily overwritten by the communicative meaning of the displayed gaze (Landes et al., 2016). According to Bayliss et al. (2006), the increase in the desirability of objects that are being looked at derives from the implicit assumption of the viewer that gaze direction indicates preference. Thus, observing another person looking at an object may lead to the conclusion that the person giving the cue likes the object he or she is looking at, which consequently results in a mimetic desire for that same object (Bry et al., 2011). If consumers have in fact already formed a preference for a product on the basis of the product’s characteristics, this preference might be less malleable by an observed gaze. However, besides implying that the gazer likes the object, a gaze that is turned toward an object can also indicate that the gazing person intends to interact with the object (Frischen et al., 2007), thus reflecting a motivational rather than an evaluative intention. In fact, research suggests that even though liking and wanting are related constructs, they might still be dissociated (e.g., Finlayson et al., 2007; Garbinsky et al., 2014) in the sense that liking is an affective state that does not necessarily include the motivation to act upon an object. It is likely that in our study, consumers perceived gaze as an indication of the intention to engage with the product rather than an evaluative signal. Thus, gaze direction influenced only their intentions to purchase the product, which is a motivational rather than an evaluative outcome. Clearly, further research is needed to examine the meaning of social gaze in an advertisement context.

### Limitations and Future Research

Due to the limited sample size and the complexity of our methodological approach the present study did not consider possible gender differences in consumers’ attention to and evaluations of banner advertisements that feature a human face. A further examination of gender effects is, however, interesting for two main reasons. First, research indicates that, in general, men and women apply different processing strategies when evaluating advertisements and their content. Whereas men tend to rely on objective information in advertisements (i.e., tangible features that are directly related to the advertised product) women engage in more comprehensive processing of both the objective and the subjective elements of an advertisement paying more attention to subtle information that is not a direct feature of the advertised product (Meyers-Levy and Maheswaran, 1991; Darley and Smith, 1995). As such a model’s face and gaze may be attended to and considered more strongly by female consumers. Second, previous research revealed differences in men’s and women’s sensitivity to social information with women usually displaying stronger reactions to emotional stimuli (Lithari et al., 2010; Thompson and Voyer, 2014), a quicker and more accurate identification of facial expressions (McBain et al., 2009; Donges et al., 2012) and a stronger empathy and tendency to mimic the facial expressions of others (Stel and van Knippenberg, 2008). They moreover attend more to informative social cues, such as another person’s eyes (Hall et al., 2010), and show stronger reflexive shifts in attention following another person’s gaze movement (Bayliss et al., 2005). Since researchers have argued that gaze cueing effects on attention allocation and object evaluations do not solely rely on a low level system of attention orientation but involve the social processing of gaze information (Bayliss et al., 2005; Bry et al., 2011), the impact of human faces in banner advertisements may be stronger in women who show a higher sensitivity for social information and a distinguished ability to mimic others’ expressions. To examine this notion, future studies will have to systematically compare participants’ gender and vary the gender of the depicted model to additionally test for effects of similarities between the viewer and the advertising model.

Besides addressing the gender of the viewer and the model, future research may also focus on the facial expression of the advertising model. While gaze cueing effects on attention are in general not affected by the facial expression of the gazing face (e.g., Hietanen and Leppänen, 2003; Bayliss et al., 2007; for a recent exception, see Niedźwiecka and Tomalski, 2015) this does not hold for the effect of facial expression on the affective response to gazed at objects which are usually evaluated most positively when the face displays a positive facial expression as compared to a neutral expression or expressions of anger or disgust (Bayliss et al., 2007). Presenting facial expressions that are slightly negative or neutral is not uncommon in advertisements and it is yet unclear how consumers react to these less positive demonstrations of emotions. A useful tool to assess consumers’ affective response to banner advertisements which display faces with different facial expressions is provided with the ability of eye-tracking systems to record consumers’ pupil size variations as a physiological measure of the affective processing of looked-at stimuli (Serfas et al., 2014; Palcu and Florack, 2016). Such measures could offer interesting insights into whether the effect of directed gaze on product evaluations is driven by a transfer of a positive affective reaction to a smiling human face onto an advertised product.

Finally, the current study applied only one banner advertisement involving one model and one product category mainly targeting female consumers. To test the generalizability of our results additional replications of the gaze cueing effect in banner advertisement are needed which should vary the contextual factors of the advertisement (e.g., type of webpage, advertisement size and position) as well as the advertisement content (e.g., category of the advertised product). Given that our study is the first to demonstrate an effect of gaze direction on the evaluation of gazed at products in an advertising context more research is needed to assess the validity of our findings and the conditions under which this effect occurs. Moreover, we took only measures of visual attention and product evaluation into account. While visual attention constitutes a prerequisite for the perception of an advertisement’s message, the investigation of further measures is advisable. In line with the objectives of online marketing, this particularly includes the click-through rate, the evaluation of the advertisement and brand as well as memory performance with respect to the advertised content. One outcome variable that warrants additional consideration is the willingness to pay for the advertised product. It is surprising that, in our study, purchase intentions were affected by the perceived gaze direction of the face while the willingness to pay was not. Event though purchase intentions and the willingness to pay for a product are related consumers frequently experience difficulties to state their true willingness to pay in hypothetical scenarios (Wertenbroch and Skiera, 2002). Alternative methods, such as incentive-based approaches in which participants are obliged to purchase a product for a price they themselves indicate (Wertenbroch and Skiera, 2002) may be more suited to assess consumers’ actual willingness to pay for an advertised product that is presented with an advertising model who is displaying different gaze directions.

## Conclusion

From a theoretical perspective, the present study provides evidence that the gaze cuing effect can be applied to banner advertising. For practical application, the present findings demonstrate that banner advertisements featuring a human face are not *per se* advantageous over banners that simply display the product. When advertisements feature social stimuli, animation can be effective for attracting viewers’ visual attention and increasing their engagement with the advertisement. This alone, however, does not ensure that observers will also engage more with the advertised product. In this respect, directed gaze cues proved effective at turning viewers’ attention toward the product region of the advertisement. However, engagement with the advertised product was not influenced by the gaze direction that was depicted. Therefore, while gaze cuing appears to constitute an adequate tool for directing attention toward important contents in an advertisement, the factors that lead to an increased processing of the content that is being gazed at need to be established in future research.

Most important, our results demonstrate an effect of gaze direction on consumers’ intention to purchase a product but not on their evaluation of the product. This suggests that gaze cuing mainly influences consumers’ intention to engage with the product rather than the extent to which they like the product.

## Author Contributions

JP and JS designed the study. JS conducted the study. JP, JS, and AF conducted the literature review and wrote the research summaries. JP and JS analyzed the data. JP and JS wrote the first draft of the manuscript, and all authors contributed to and have approved the final manuscript. Authors had full access to the study data.

## Conflict of Interest Statement

The authors declare that the research was conducted in the absence of any commercial or financial relationships that could be construed as a potential conflict of interest. The reviewer CH and handling Editor declared their shared affiliation, and the handling Editor states that the process nevertheless met the standards of a fair and objective review.
